# *DPYD* Exome, mRNA Expression and Uracil Levels in Early Severe Toxicity to Fluoropyrimidines: An Extreme Phenotype Approach

**DOI:** 10.3390/jpm11080792

**Published:** 2021-08-13

**Authors:** Priscila Villalvazo, Belén Marzal-Alfaro, Pilar García-Alfonso, José Luis Revuelta-Herrero, Fabienne Thomas, Sara López-Tarruella, Xandra García-González, Aitana Calvo, Malika Yakoubi, Sara Salvador-Martín, Flora López-López, Iker Aguilar, María Sanjurjo-Sáez, Miguel Martín, Luis Andrés López-Fernández

**Affiliations:** 1 Servicio de Farmacia, Instituto de Investigación Sanitaria Gregorio Marañón, Hospital General Universitario Gregorio Marañón, 28007 Madrid, Spain; pvillalv@ucm.es (P.V.); belen.marzal@salud.madrid.org (B.M.-A.); joseluis.revuelta@salud.madrid.org (J.L.R.-H.); xandra.garcia@salud.madrid.org (X.G.-G.); sara.salvador@iisgm.com (S.S.-M.); maria.sanjurjo@salud.madrid.org (M.S.-S.); 2 Servicio de Oncología Médica, Instituto de Investigación Sanitaria Gregorio Marañón, CiberOnc, Hospital General Universitario Gregorio Marañón, Universidad Complutense de Madrid, 28007 Madrid, Spain; pgarcaalfonso@gmail.com (P.G.-A.); slopeztarruella@gmail.com (S.L.-T.); pilaraitana.calvo@salud.madrid.org (A.C.); mmartin@geicam.org (M.M.); 3 Department of Pharmacology, Institut Claudius-Regaud, CRCT, Université de Toulouse, Inserm, UPS, 20-24, 31300 Toulouse, France; ThomasJean.Fabienne@iuct-oncopole.fr (F.T.); yokoubi.malika@iuct-omcopole.fr (M.Y.); 4 Servicio de Oncología Médica, Fundación de Investigación Sanitaria Doce de Octubre, Hospital Universitario Doce de Octubre, 28041 Madrid, Spain; flora.lopez@salud.madrid.org; 5 Servicio de Oncología Médica, Instituto de Investigación Sanitaria Ramón y Cajal, Hospital Universitario Ramón y Cajal, 28034 Madrid, Spain; Iker.aguilar@salud.madrid.org

**Keywords:** pharmacogenetics, cancer, adverse drug events, capecitabine, 5-fluorouracil

## Abstract

Dihydropyrimidine dehydrogenase deficiency is a major cause of severe fluoropyrimidine-induced toxicity and could lead to interruption of chemotherapy or life-threatening adverse reactions. This study aimed to characterize the *DPYD* exon sequence, mRNA expression and in vivo DPD activity by plasma uracil concentration. It was carried out in two groups of patients with extreme phenotypes (toxicity versus control) newly treated with a fluoropyrimidine, during the first three cycles of treatment. A novel nonsense gene variant (c.2197insA) was most likely responsible for fluoropyrimidine-induced toxicity in one patient, while neither *DPYD* mRNA expression nor plasma uracil concentration was globally associated with early toxicity. Our present work may help improve pharmacogenetic testing to avoid severe and undesirable adverse reactions to fluoropyrimidine treatment and it also supports the idea of looking beyond *DPYD*.

## 1. Introduction

Severe adverse reactions to fluoropyrimidines are some of the main problems related to chemotherapy treatment in solid cancers [1]. These reactions occur in about one in five patients [2]. Dihydropyrimidine dehydrogenase (DPD) is the key metabolizer of 5-fluorouracil. Partial DPD deficiency is estimated in 3–15% of the Caucasian population, and 0.1–0.5% have a complete deficiency [3,4]. The relationship between DPD deficiency and the increase in fluoropyrimidine-induced toxicity in patients who take 5-fluorouracil or its prodrug capecitabine is well established [5]. These undesirable adverse reactions may compromise treatment outcomes, due to delays in administration, dose reductions and even necessary withdrawals of the drug.

Multiple factors, such as age and sex, are associated with severe adverse reactions to fluoropyrimidines and have been used for therapy individualization [6,7]. However, genetic factors, mainly in the *DPYD* gene, have been revealed as the most common cause of severe and life-threatening events induced by fluoropyrimidines [8]. Several drug regulatory agencies recommend genotyping of some variants in *DPYD* or phenotyping of DPD activity before starting treatment with fluoropyrimidines [9].

At least four variants are widely recommended for genotyping prior to the first administration of fluoropyrimidines, due to their well-established relationship with toxicity [10]. These variants include c.1905 + 1G > A (rs3918290), which causes the exon 14 deletion, two missense variants, c.1679T>G (rs55886062) and c.2846A>T (rs67376798), and the intron variant c.1129–5923 (rs75017182), which causes a splice defect. Although the incorporation of this test into clinical practice can avoid 25–50% of severe adverse reactions, there is still room for improvement [11,12]. Other rare genetic variants of *DPYD* are also clearly associated with fluoropyrimidine-induced toxicity and many others are probably still unknown [13]. In addition, new variants have been identified after widespread *DPYD* genetic testing in clinical practice [14,15]. Furthermore, some patients without any genetic variant of *DPYD* can suffer severe adverse reactions to fluoropyrimidines [14], which suggest that other factors must play a role.

Variation in copy number of intragenic regions has also been described as affecting DPD functionality [16]. Recently, a novel intragenic deletion in *DPYD* which includes exon 4 was found with a high prevalence in Finnish cancer patients receiving capecitabine. This deletion causes a defect in the splicing and thus the loss of exon 4, leading to the generation of a truncated DPD (p.Cys79Thrfs*8).

DPD phenotyping is an alternative to *DPYD* genotyping. DPD participates in the conversion of uracil (U) to dihydrouracil (UH2). The European Medicine Agency (EMA) recommends DPD testing prior to treatment with fluoropyrimidines [17]. Meanwhile, the Spanish Medicine Agency (AEMPS) established that plasma U levels of 16–150 ng/mL correspond to a partial deficiency and levels above 150 ng/mL indicate a complete deficiency [18].

Another mechanism of DPD activity variation and changes in risk induced by fluoropyrimidines is thorough transcriptional regulation of *DPYD*. Epigenetic alterations may affect *DPYD* expression, as shown in the RKO colorectal cancer cell line [19]. These changes in *DPYD* mRNA expression could affect the quantity of DPD generated in some tissues and potentially correlate with fluoropyrimidine-induced toxicity.

The approach of genotyping patients with an extreme phenotype has been shown to be useful in pharmacogenetics to identify new biomarkers [20,21]. In this work, we compare the whole exon sequence of the *DPYD* gene, DPD activity and *DPYD* mRNA expression in patients with toxicity grade ≥3 during the first three cycles versus a control cohort with toxicity <3 during at least eight cycles of fluoropyrimidine treatment.

## 2. Materials and Methods

### 2.1. Patients

This was a retrospective, observational, longitudinal case–control study. It included patients aged ≥ 18 years, diagnosed with a solid tumor and newly treated with a chemotherapy regime based on fluoropyrimidines. An exhaustive review of 464 medical records from patients treated with fluoropyrimidines since January of 2018 was carried out to select two extreme phenotypes. First, 28 patients presenting severe adverse reactions (grade ≥ 3 following the CTCAE v5 classification) during the three first cycles of fluoropyrimidine-based treatment and, second, 14 patients who did not present any toxicity grade ≤ 1 during at least eight cycles of treatment. The previous periods were the minimum to classify the patients in one of the two groups. Patients were recruited from January 2020 to March 2021. In our hospital, all patients are usually genotyped for c.1905 + 1G > A (rs3918290), c.1679T>G (rs55886062), c.2846A>T (rs67376798) and c.1129–5923 (rs75017182) prior to the first administration of the fluoropyrimidine. Those participants who were not previously genotyped for these variants were screened and, if positive, excluded from the study.

The following clinical and demographic variables were recorded: age, sex, fluoropyrimidine used and concomitant medication and type of cancer.

Adverse events during every cycle were compiled from the hospital’s medical records. These included GI disorders such as diarrhea, nausea and/or vomiting; hand–foot syndrome, mucositis, hematologic toxicity and other biochemical parameters. Classification and severity rates were based on National Cancer Institute common terminology criteria for adverse events v5.

### 2.2. Ethics

This study was approved by the Institutional Ethics Committee of Hospital Gregorio Marañón (protocol code FG-2019-02, date of approval 23 September 2019). All patients signed a written informed consent.

### 2.3. DPYD Exon Sequencing

Genomic DNA was isolated from whole blood using a QIAamp DNA Blood Mini Kit following the manufacturer’s instructions (Qiagen, Hiden, Germay). All of the 23 exons from the *DPYD* gene and flanking intron regions were amplified as described in García-González et al. [15]. PCR products (5 µL) were purified using PureIT ExoZAP (2 µL) (Ampliqon, Odense, Denmark). Sequences were obtained at the Genomics Unit of Hospital Gregorio Marañón by Sanger technology and analyzed using SnapGene v5.2. DPD activity was predicted from genotype using SIFT, Polyphen2 and ClinVar [22,23,24]. Linkage disequilibrium was measured using the LDmatrix tool (National Cancer Institute) [25].

### 2.4. DPD Activity Measure

Blood samples were collected from patients in EDTA tubes between 8:00 a.m. and 11:00 a.m. Plasma was isolated by centrifugation at 3000 r.p.m. for 10 minutes. U concentration was measured in all patients as described previously [4]. A patient was catalogued as partially deficient if U > 16.

### 2.5. DPYD mRNA Expression

#### 2.5.1. RNA Isolation cDNA Synthesis

Total RNA was isolated from whole blood and preserved in PAXgene^®^ tubes using a PAXgene™ Blood RNA kit (PreAnalytics, Hombrechtikon, Switzerland). Complementary DNA (cDNA) was synthetized from 300 ng of total RNA using a High-Capacity cDNA Reverse Transcription kit (Applied Biosystems, Foster City, CA, USA) in a final volume of 20 µL using random hexamers. A 1/10 dilution was used in real-time PCR. All of these procedures were performed as recommended by the manufacturers.

#### 2.5.2. Real-Time PCR and Quantification Method

*DPYD* was relatively quantified by RT-PCR in a StepOne (Applied Biosystems, Foster City, CA, USA) using *HPRT1* for normalization. RT-PCR was performed in triplicate using the following TaqMan probes: *DPYD* (Hs115750_m1) and *HPRT1* (Hs02800695_m1) (Applied Biosystems, Waltham, MA, USA) in 20µL of final volume (10 µL Universal Master Mix, Applied Biosystems; 2µL cDNA 1/10 dilution; 1µlL TaqMan probe, 7µL free-nuclease water). Relative *DPYD* expression in toxicity versus control group was quantified using the 2^−∆∆Ct^ method, taking as relative expression the sample D031, and in StepOnePlus v-2.3.2 (Applied Biosystems). Relative values of mRNA *DPYD* expression were represented in Prism version 8 (San Diego, CA, USA).

### 2.6. Characterization of 2197insA

#### 2.6.1. mRNA Sequencing

Blood was collected in Paxgene Blood RNA tubes (Becton Dickinson, Franklin Lakes, NJ, USA) and RNA isolated using a PaxGene RNA blood RNA kit (Qiagen, Valencia, CA, USA). cDNA was synthesized and diluted as described in Section 2.5.1. The cDNA region containing the 2197insA was amplified in 10 µL (NZY Master Mix 5 µL, 1 µM of primer 17F2 (5’-TGA GCA TCG CAA GAG CTG CA), 1 µM of primer Sp20R (5’ TGG ACT CTG TCC ATC CCA GTC T) and 10 ng of genomic DNA). Sequences were obtained in the Genomics Unit of our hospital using primer Sp20R as a template.

#### 2.6.2. 3D Modeling

The DPD sequence generated by 2197insA and wild type DPD were exported from SnapGene v5.2 in Fasta format. The 3D modeling of wild type DPD and DPD 2197insA was carried out using the RaptorX structure prediction server [26]. Protein database files were downloaded and visualized using Chimera [27].

### 2.7. Analysis of Exon 4 Skipping

The cDNA region from exon E1-2 to E5 was amplified in all participants to identify the loss of exon 4. PCR amplifications were performed in 10 µL (NZY Master Mix 5 µL, 1 µM of primer E1-2-F (5’-TCG GCG GAC ATC GAG AGT AT), 1 µM of primer E5-R (5’ AGA GGT TGG ACA TAC CAT TCC A) and 2µL of 1/20 cDNA dilution). PCR was performed with the following conditions: 94 °C 8 min, 40 cycles of 94 °C 30 s, 60 °C 30 s, 72 °C 1 min and a final step of 72 °C 5 min. Fragment length was analyzed using a DNA1000 kit in a 2100 Bioanalyzer (Agilent Technologies, Santa Clara, CA, USA). A fragment of 375 nucleotides in length was expected for wild type individuals and a fragment of 287 nucleotides for individuals who skip exon 4.

## 3. Results

### 3.1. Clinical and Demographic Characteristics of Patients

After review of 464 clinical records, 41 patients met the inclusion criteria. They were separated into two groups: 14 patients were included in the control group (fluoropyrimidine-related adverse reactions ≤ 1) and 28 patients in the toxicity group (adverse reactions grade ≥3 during the first three cycles). Characteristics of participants are summarized in Table 1. No statistical differences were observed in gender, age, type of cancer, clinical stage, surgery or concomitant chemotherapy. However, the control group included more capecitabine-treated patients (85% vs. 48.1%) and, as expected, due to classification of the groups, a higher fluoropyrimidine cumulative dose, and a lower need for dose reductions due to toxicity after the first cycle (*p* value < 0.05).

Table 2 shows the incidence of adverse events <2 and ≥3. As expected, all adverse reactions of any degree were more common in the toxicity group, but the difference between groups was statistically significant only for leukopenia, neutropenia, lymphopenia, diarrhea and nausea/vomiting.

### 3.2. DPYD Genetic Variants in Coding Regions and Splice Sites

**Figure 1:**
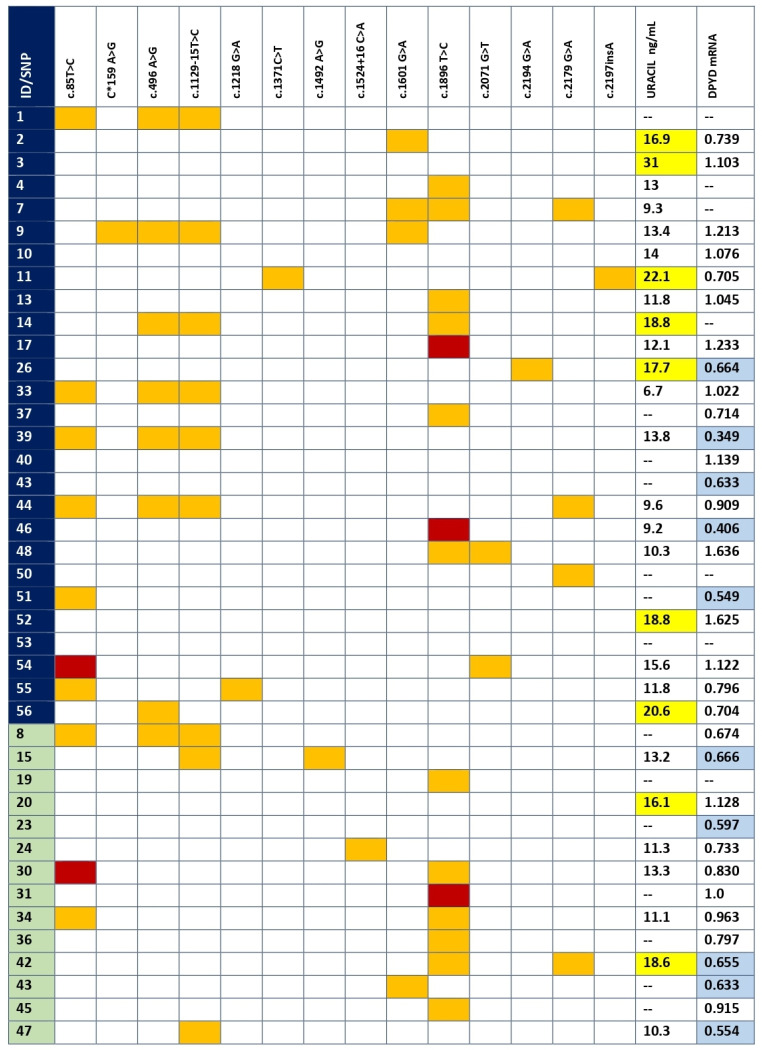
SNPs, mRNA expression and DPD activity per patient. SNPs found in coding and flanking intronic regions for each patient. Dark blue, toxicity group; green, control group; orange, heterozygous; red, homozygous mutant; yellow, U > 16 ng/mL; light blue, below 25th percentile in mRNA expression. The variant c*159A>G is a new variant of unknown significance placed in the 3′UTR.

The whole *DPYD* sequencing of the coding exon and flanking intron regions (20 bp on both sides) in the 41 patients revealed the presence of 14 SNPs: three synonymous, eight non-synonymous (seven missense and one nonsense), one in the 3′ untranslated region (UTR) and two in the intron region close to an exon. The variants found in every patient can be found in Figure 1. No great differences were observed, in the number of SNPs (12 vs. 8), in SNPs per patient (1.62 vs. 1.43) or in the number of patients without any SNP in the *DPYD* gene between controls and patients with early severe toxicity (six vs. two, respectively).

The SNPs were categorized into three groups: specific to the toxicity group, shared by toxicity and control groups and specific to the control group (Table 3). The most relevant variants found were two new SNPs that were specific to the patients with toxicity. The variant c.2197insA contains an insertion that provokes amino acid changes in 14 positions until the arrival of a stop codon, consequently generating a shortened DPD (p.Thr733AsnfsTer14). The variant c*159A>G is a new variant of unknown significance placed in the 3′UTR. Another two very infrequent SNPs were found in the toxicity group (p.Met406Ile and p.Val691Leu), both with controversial results from several information sources, such as the Clinical Variation Consortium, Sort Intolerant from Tolerant (SIFT) and Polymorphism Phenotyping (PolyPhen) databases.

Regarding the SNPs found both in the control and the toxicity groups, six variants were found but only one was more frequent in the toxicity group compared to the control group, p.Met166Val (25.9% vs. 7.1%, respectively). However, this increase was not statistically significant. Interestingly, the haplotype c.496G/c.1129-15C was more frequently observed in the toxicity group (25.9%) than in the control one (7.1%). Both SNPs are in disequilibrium linkage (D′ = 0.955, R^2^ = 0.911). This analysis was also performed by tumor type and no statistically significant association was obtained (Supplementary Table S1).

**Figure 2:**
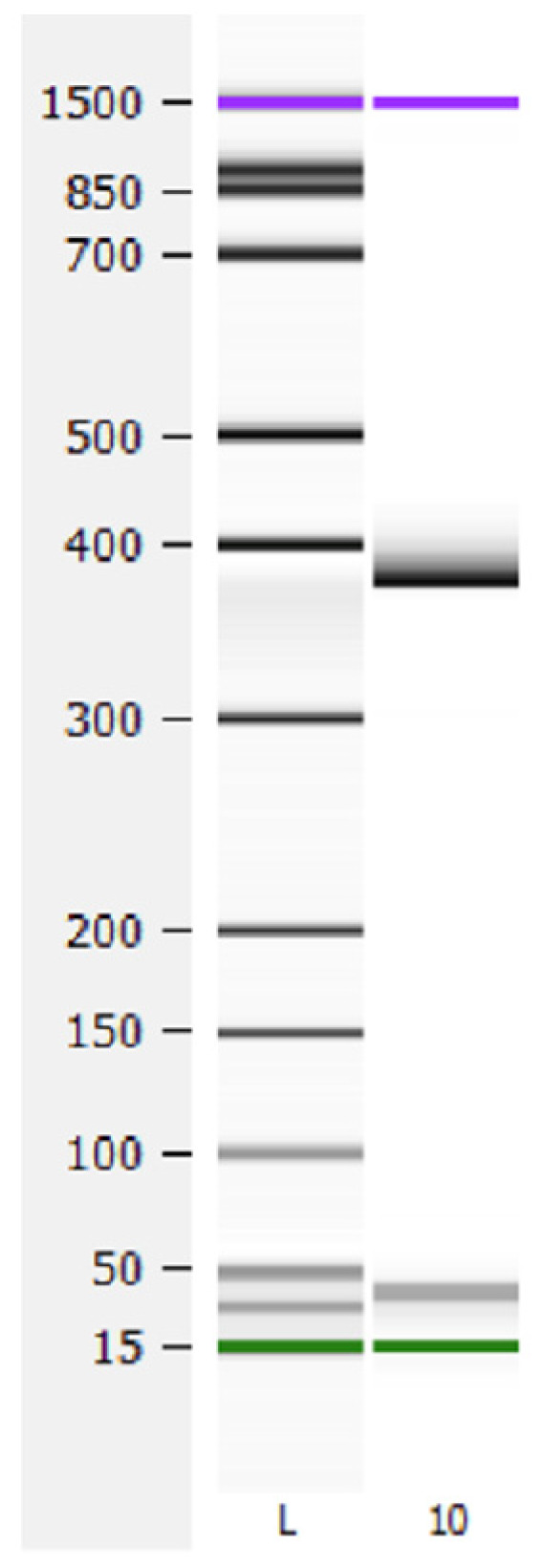
Analysis of exon 4 skipping. cDNA from exon 1 to 5 was amplified. A wild type fragment of 375 nucleotides was observed in all samples. The expected fragment of 287 nucleotides resulting from exon 4 skipping was not detected in any sample. L, ladder; 10, patient 10.

Exon 4 skipping was not detected in any patient after analysis of PCR length fragment from cDNA using oligonucleotides placed in exons 1 and 5. A fragment of 375 nucleotides containing exon 4 was amplified for all patients, while the expected 287 nucleotide fragment generated after exclusion of exon 4 was not found in any sample (Figure 2).

### 3.3. DPD Activity by Uracil Concentration

**Figure 3:**
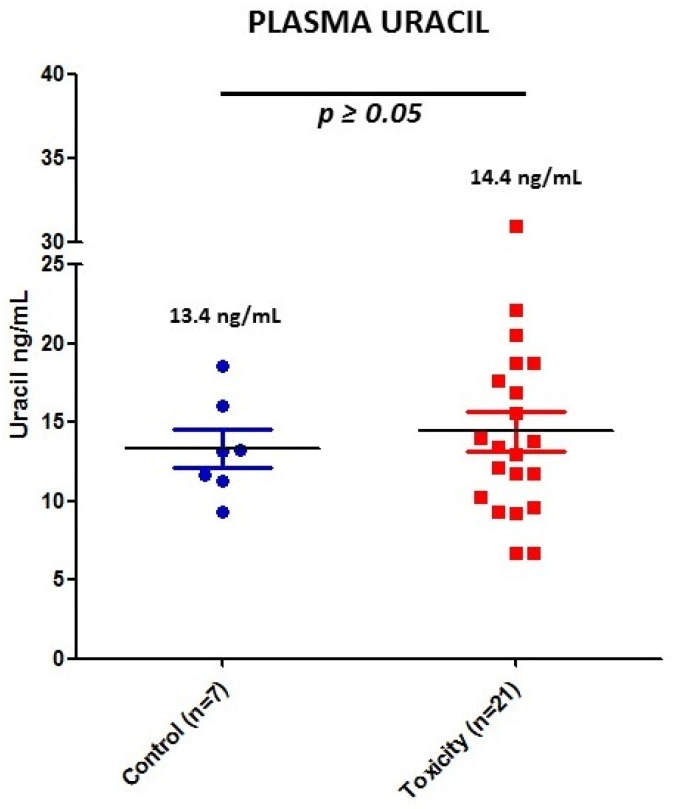
Plasma U concentrations for the 28 patients with validated measurement of DPD activity. Blue, control patients; red, early toxicity patients.

DPD activity was indirectly measured for each patient by quantifying U concentration (see individual values in Figure 1). DPD activity was measured in seven controls and 21 patients of the severe toxicity group. Since DPD activity is dependent on circadian rhythms, seven samples from the control group and six from the toxicity group were discarded because they were collected after 11:00 a.m. Seven patients in the toxicity group (2, 3, 11, 14, 26, 52 and 56) had U > 16 and were classified as DPD deficient, while two patients (20 and 42) from the control group also had U > 16. However, the analysis did not show differences in U between control and severe toxicity groups. Mean plasma U was 14.4 ng/mL (IQR 8.3, 6.7–31.0) in the toxicity group and 13.4 ng/mL (IQR 4.8, 9.8–18.6) in the control group (*p* value > 0.05) (Figure 3).

### 3.4. mRNA DPYD Expression

**Figure 4:**
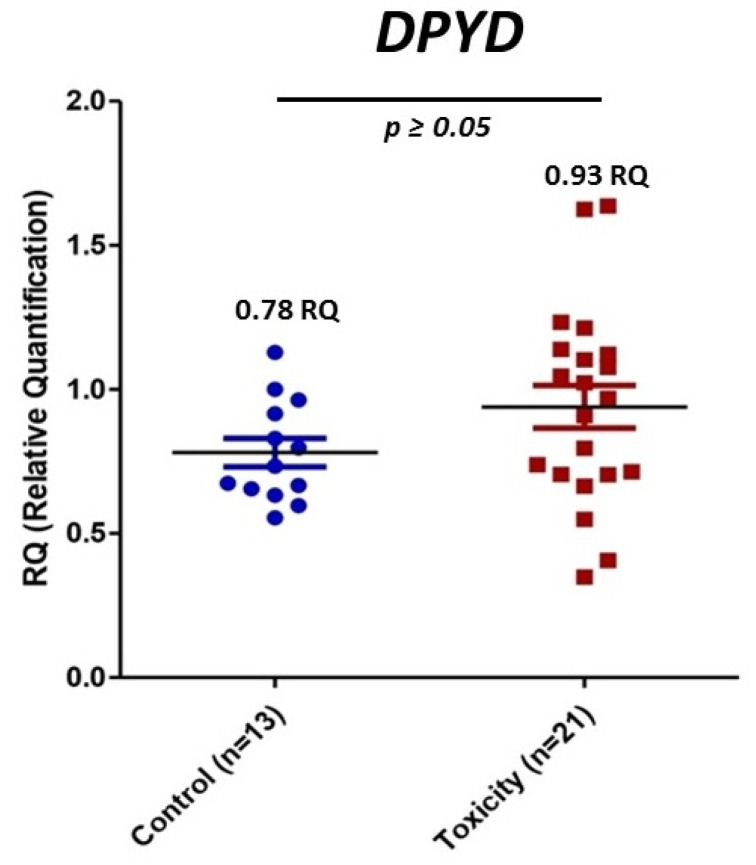
Relative mRNA expression of *DPYD* in controls and early severe toxicity patients. Blue, control patients; red, early toxicity patients.

PaxGene tubes were collected from all the recruited patients. However, total RNA isolation was not high enough quality in 12 samples. We obtained a new sample for RNA isolation in five patients, but seven declined to provide a new sample or had died. The expression of mRNA of *DPYD* was measured in 13 controls and 21 patients of the early severe toxicity group (see individual values in Figure 1). The analysis did not show differential expression (*p* value > 0.05) of *DPYD* mRNA between controls and patients with early severe toxicity (Figure 4).

### 3.5. Characterization of c.2197insA

#### 3.5.1. Sequence Analysis

**Figure 5:**
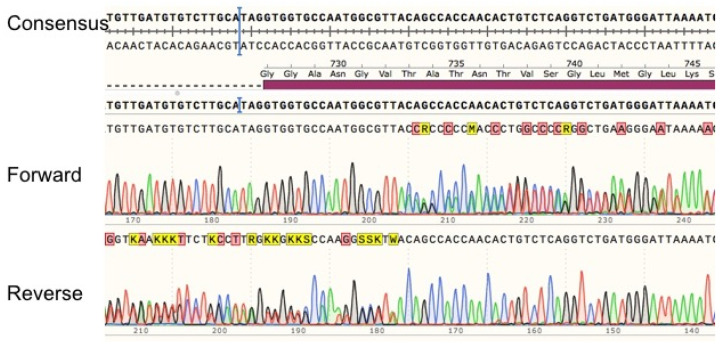
Sequencing alignment of exon 18 for patient with c.2197insA. Exon 18 PCR amplification was sequenced using forward and reverse primers for this exon. Sequences were aligned with the consensus sequence (NC_000001.11 GRCh38.p13).

Patient 11 showed an insertion in position 2197 after the starting codon. Sequencing of exon 18 for this patient showed a clean sequence until this position using a forward primer as well as a reverse primer (Figure 5). The read of duplicated peaks shows an insertion of an adenine at this position.

The insertion of an A into the *DPYD* mRNA was verified by sequencing the complementary DNA from whole blood of this patient.

#### 3.5.2. DPD Modeling Generated by c.2197insA Variant

**Figure 6:**
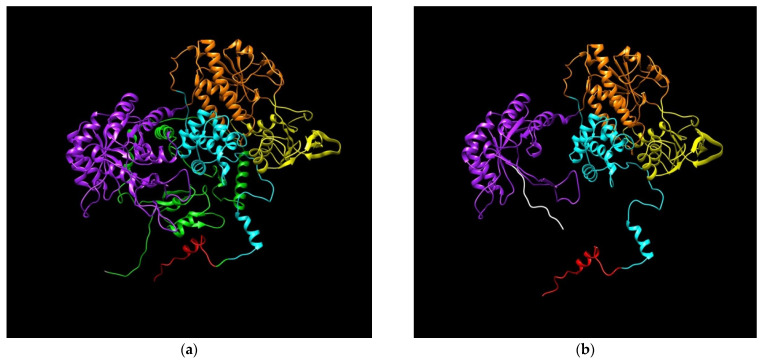
Predicted models for DPD. (**a**) Wild type DPD; (**b**) DPD generated by insA in c.2197. Protein Database (PDB) files were generated using RaptorX web software (raptorX.uchicago.edu) and images were obtained using Chimera v1.13.1. DPD domains: I, N-terminal Fe-S clusters (red); II, flavin mononucleotide (FAD) binding (orange); III, NADPH binding (yellow); IV, flavin adenine dinucleotide (FMN)/pyrimidine binding (purple); and V, C-terminal Fe-S clusters (green). Amino acids added from insertion to stop codon (white).

The adenine insertion at position 2197 of *DPYD* mRNA after the ATG initiation predicts the generation of a truncated protein of 746 amino acids. The truncated DPD protein sequence is the same until amino acid position 732, different from position 733 to 746 and lacks the fragment from 747 to the end (position 1225). This anomaly of DPD breaks the flavin mononucleotide domain, where the pyrimidine binding site is located, and lacks the 4Fe-4S domain. Results of 3D modeling of mutated and wild type DPD were obtained using the RaptorX web server and colored and then visualized using Chimera (Figure 6). Clear differences are shown in the central structure of DPD affecting the missing domains (Supplementary Materials Video S1).

According to plasma U concentration, the patient carrying this mutation showed a partial DPD deficit (U = 22.1 ng/mL).

## 4. Discussion

The use of the whole coding *DPYD* sequence followed by measurement of DPD activity is an efficient approach to identify new pathogenic genetic variants [14,15,16,28,29]. However, this approach is not able to explain all severe fluoropyrimidine-induced toxicities. In this work, we compared genetic variants, protein activity and blood *DPYD* mRNA expression in 41 cancer patients receiving fluoropyrimidine-based treatment. We have separated them in two groups: those that suffered early severe toxicity (cycles 1–3) and those that did not.

Our study has disclosed and characterized c.2197insA, a new and infrequent genetic variant most likely associated with DPD deficiency and early severe toxicity to fluoropyrimidine-based treatments. The predicted human DPD model showed that the sequence carrying variant c.2197insA generates a truncated protein (p.Thr733AsnfsTer14) affecting the flavin mononucleotide/pyrimidine binding domain and the C-terminal Fe-S clusters [30]. DPD works as a homodimer and these alterations clearly suggest that the truncated protein is not able to bind 5-FU, impeding drug metabolism. Measurement of plasma U levels showed that the patient carrying this mutation was DPD deficient. Accumulation of 5-FU due to inefficient elimination is probably the cause of severe toxicity in the patient carrying this mutation. Another SNP (c.2242+1G>T) leads to skipping of exon 19 and generates a very similar truncated DPD protein which lacks the same domains [15]. This variant was associated with capecitabine-induced severe toxicity in a breast cancer patient. According to this evidence, the presence of c.2197insA is the most probable cause of the toxicity observed in the carrier patient.

A SNP in the position c*159A>G in the 3′UTR was identified in patient 9. Variants in the 3′UTR may affect mRNA stability, translation efficiency, nuclear export and cellular location [31]. This variant does not seem to affect the polyadenylation signal region, one of the most sensitive places in the 3′UTR [32]. However, it may affect microRNA binding sites. Expression of *DPYD* mRNA in this patient was low in comparison with other samples. No 3′UTR *DPYD* variants have been associated with toxicity to fluoropyrimidines to date. Nevertheless, a study analyzing 33 germline polymorphisms in the 3′UTR of genes involved in drug absorption, distribution, metabolism and elimination (ADME) showed that *DPYD* rs291593 was associated with recurrence-free survival in breast cancer patients [33]. No data on the effect of this SNP on *DPYD* expression have been reported, but the variant position suggests a putative posttranscriptional regulation, a role that would explain its relationship with treatment response. In a similar way, c*159A>G could alter *DPYD* mRNA stability and decrease DPD translation, leading to the early toxicity observed in this patient. The low frequency of this variant hampers the chance to prove this relationship in bigger cohort studies.

Another two non-synonymous genetic variants were only found in the group of patients with early severe toxicity to fluoropyrimidines, p.Met406Ile (patient 55) and p.Val691Leu (patients 48 and 54). p.Met406Ile was categorized as benign by SIFT and tolerated by PolyPhen, but ClinVar recognized a conflicting interpretation. No damaging effect of this SNP on DPD activity has been reported [34]. Accordingly, patient 55 had U and *DPYD* mRNA expression levels that were within range. pVal691Leu was considered probably damaging by Polyphen-2 and tolerated by SIFT. In vitro DPD activity was measured for this SNP and considered normal [35]. Both patients carrying this variant in our study had U concentrations within the established limits of normal DPD activity. Consequently, these SNPs are not good candidates to explain the toxicity observed in the carrier patients.

Recently, a Dutch pharmacogenetics group has identified SNPs pMet166Val and c.1129-15 as fully functional with a weak level of evidence [36]. The SNP p.Met166Val was more frequent in the toxicity group than in the control group. This SNP is in linkage disequilibrium with c.1129-15C. These variants have been widely considered as not related to toxicity to fluoropyrimidines [37,38,39]. In addition, DPD in vitro activity has been reported as normal or even higher than normal for p.Met166Val [34]. However, this result is conflicting and other authors have found a relationship of this SNP with fluoropyrimidine-induced toxicity [8,40,41,42,43,44]. Furthermore, recent work showed this SNP to be associated with a lower UH_2_/U ratio, another indirect way to measure DPD activity [45]. The higher frequency of this SNP in the group of patients with early toxicity to fluoropyrimidines suggests it plays a role in the risk of toxicity. The limited sample size does not allow us to obtain conclusive results for this SNP. Nonetheless, our results suggest that the effect of haplotype c.496C/c.1129-15C on toxicity risk to fluoropyrimidines may be more relevant than previously thought and, hence, should be explored in larger studies.

Unfortunately, the approach followed in this work was not able to explain why most of the recruited patients suffered early severe toxicity as a consequence of their fluoropyrimidine-based treatment. Recently, in a similar study, the skipping of exon 4 was found to be common in patients from Finland with severe toxicity to fluoropyrimidines [16]. This established a promising biomarker to help increase the power of detection of risky patients of severe toxicity induced by fluoropyrimidines. However, none of the patients included in our study had mRNA skipping of exon 4. Thus, the clinical usefulness of this biomarker outside of the Finnish population remains to be elucidated.

High *DPYD* mRNA expression correlates with poor disease-free survival [46]. Our group observed lower *DPYD* mRNA expression (data not shown) in descendants of a breast cancer patient suffering from severe toxicity to capecitabine and carrying a genetic variant in *DPYD* skipping of exon 19 [15]. In this study, we analyzed *DPYD* mRNA expression in control and toxicity groups of patients treated with fluoropyrimidines. *DPYD* mRNA expression was similar in control and toxicity groups and failed to identify patients at risk of severe fluoropyrimidine toxicity. On the one hand, previous studies found a strong correlation between *DPYD* mRNA expression with DPD activity in liver sections [47] and in tumor tissue [48]. On the other hand, it has been suggested that *DPYD* mRNA might not be reflective of global DPD activity, because it does not distinguish mRNA coding for non-functional DPD [49]. *DPYD* expression is regulated by *STAT3* [50], interferon alpha [51], *TWIST1* [52] and several microRNAs or long non-coding RNAs [53,54]. None of these genes have been associated with toxicity to fluoropyrimidines. A limitation of this part of our study is that samples for mRNA expression were collected after patients were classified in the control or toxicity group and, therefore, had already received several cycles of chemotherapy. Perhaps collecting samples prior to the start of therapy would have rendered more significant results. More and larger studies are needed to explore the effect of other variables, such as age and sex.

Only one of the newly discovered variants in the toxicity group, c.2197insA, seemed to be clearly associated with DPD deficiency and early toxicity, based on DPD modeling and the patient’s level of plasma U. Furthermore, the absence of inactivating mutations is not the only mechanism involved in DPD activity, for example, methylation has been recognized as a gene silencing mechanism [55]. Ezzeldin et al. found that 100% of patients with DPD deficiency without inactivating mutations in the *DPYD* gene had aberrant methylation of the *DPYD* promoter [56]. Moreover, no correlation was observed between U or UH_2_/U and *DPYD* mRNA expression, suggesting that DPD activity is not dependent on gene expression in whole blood.

Interestingly, two patients with a DPD-deficient phenotype, as established by plasmatic U in the toxicity group (patients 3 and 52) and one in the control group (patient 20), did not carry any *DPYD* variant. Intriguingly, no *DPYD* variants were observed in the patient with the greatest activity deficit (patient 3, U = 31 ng/mL) and their *DPYD* mRNA expression was above the 25th percentile. Intriguingly, patient 42 in the control group showed partial DPD deficiency by plasma U and presented low *DPYD* mRNA expression.

The measurement of plasma U is widely accepted for detecting DPD deficiency [4,57]. However, our results do not show a clear association of U with the occurrence of early severe fluoropyrimidine toxicity. It has been recently reported that an artificial increase in U concentration during fluoropyrimidine treatment can lead to DPD deficiency misinterpretation [58]. Competition of U and 5-fluorouracil for DPD may increase U plasma concentration. However, 5-FU was detected only in one patient (data not shown).

Altogether, the present findings strongly suggest that several other variables may influence the tolerability of fluoropyrimidine-based treatments. Nevertheless, a larger comparative case–control study involving thousands of individuals is needed to draw statistically significant conclusions. In addition, these results support the use of approaches including multiple genes and other parameters in future studies in order to try to better predict fluoropyrimidine-induced toxicity.

### Future Research Directions

We used a multiple approach, studying *DPYD* genetic variants, mRNA expression and indirect measuring of DPD activity to try to identify the cause of severe fluoropyrimidine-induced toxicity in a group of cancer patients. For most of the patients, our approach was unable to provide the cause of toxicity. Other authors have used multiparametric approaches for predicting patients at risk of toxicity due to fluoropyrimidine-based treatment [59]. This seems to be the best option to explore in the future. The role of conflicting variants in the literature must be clarified to improve the prediction models. The trend observed with the haplotype c.496C/c.1129-15T and fluoropyrimidine toxicity in our study, along with recent results obtained by other groups, suggests this haplotype should be investigated in larger cohorts. Since no relationship between *DPYD* mRNA expression in whole blood and early severe toxicity has been observed in this work, in our opinion, the effort to find new biomarkers for toxicity should be focused in genotyping not only *DPYD* but also other genes, such as *TYMS*, *CDA*, *ENOSF1* and others related to fluoropyrimidine’s pharmacodynamics and pharmacokinetics, such as *CES1*, *CES2* or *UMPS*. Implementation of functional studies to prevent toxicity in those patients in whom *DPD* deficits were detected would be necessary as well.

## 5. Conclusions

The proposed multiple approach including *DPYD* gene sequencing, expression and measurement of DPD activity does not explain all the toxicity observed in fluoropyrimidine-treated patients. Thus, it is necessary to investigate other genes and factors. However, the whole *DPYD* sequencing helped us to identify a new deleterious variant, c.2197insA, that potentially codes for a non-functional DPD protein (p.Thr733AsnfsTer14) which could cause early severe toxicity.

## Figures and Tables

**Table 1:** Characteristics of patients.

Characteristic	Overall (*n* = 41)	Toxicity Group (*n* = 27)	Control Group (*n* = 14)	*p* Value
Age at diagnosis (IQR, range; years)	63 (17, 36–81)	63 (16, 36–81)	63 (19, 36–80)	0.805
Gender				
Male, *n* (%)	20	12 (42.9%)	8 (57.1%)	0.382
Female, *n* (%)	21	15 (55.5%)	6 (42.9%)	
Type of cancer				
Gastric	7	5 (18.5%)	2 (14.3%)	0.821
Rectum	7	4 (14.8%)	3 (21.4%)	
Colon	21	13 (48.1%)	8 (57.1%)	
Breast	2	2 (7.4%)	0 (0.0%)	
Pancreas	4	3 (11.1%)	1 (7.1%)	
Clinical stage				
I	0	0 (0.0%)	0 (0.0%)	0.347
IIA	2	2 (7.4%)	0 (0.0%)	
IIB	2	1 (3.7%)	1 (7.1%)	
IIIA	1	1 (3.7%)	0 (0.0%)	
IIIB	10	9 (33.3%)	1 (7.1%)	
IIIC	5	3 (11.1%)	2 (14.3%)	
IV	21	11 (40.7%)	10 (71.4%)	
Surgery, yes *n* (%)	33	23 (85.1%)	10 (71.4%)	0.292
Complete resection, yes *n* (%)	26	18 (66.6%)	8 (57.1%)	0.489
Neoadjuvant treatment, yes *n* (%)	7	4 (14.8%)	3 (21.4%)	0.321
Type of chemotherapy				
5-fluorouracil	16	14 (51.9%)	2 (14.3%)	0.025 *
Capecitabine	25	13 (48.1%)	12 (85.7%)	
Accumulated dose (g)				
5-fluorouracil	3.4 (5.0, 2.3–13.6)	3.3 (2.3, 2.3–13.6)	10.0 (NA, 9.0–11.1)	0.047 *
Capecitabine	13.1 (8.7, 2.2–16.6)	7.7 (10.3, 2.5–16.6)	13.9 (3.9, 2.2–16.6)	0.031 *
Concomitant medication				
Oxaliplatin	31	20 (74.0%)	11 (78.6%)	0.620
Irinotecan	4	2 (7.4%)	2 (14.3%)	0.457
Cetuximab	1	0 (0.0%)	1 (7.1%)	0.152
Panitumumab	2	1 (3.7%)	1 (7.1%)	0.608
Bevacizumab	5	2 (7.4%)	3 (21.4%)	0.178
Trastuzumab	2	1 (3.6%)	1 (7.1%)	0.608
Complete cycles				
1	41	27 (100%)	14 (100%)	NA
2	27	13 (48.1%)	14 (100%)	0.001 *
3	20	6 (22.2%)	14 (100%)	0.000002 *
Dose reduction due to toxicity				
Cycle 1	4	3 (11.1%)	1(7.1%)	0.709
Cycle 2	6	6 (40%)	0 (0.0%)	0.039 *
Cycle 3	7	6 (100%)	1 (7.1%)	0.002 *

IQR, interquartile range; NA, not applicable; * *p* Value < 0.05.

**Table 2:** Adverse event comparison between control and toxicity groups.

Adverse Event	Overall (*n* = 41)	Toxicity Group (*n* = 27)	Control Group (*n* = 14)	*p* Value
Hemoglobin				
Hemoglobin, g/dL #	11.9 (1.2, 7.2–14.7)	11.2 (3.1, 7.2–14.7)	12.9 (1.9, 11–13.7)	0.025 *
Anemia, *n* (%)	19 (46%)	15 (55%)	4 (28%)	0.125
Severe anemia (≥Grade 3), *n* (%)	2 (5%)	2 (7%)	0 (0%)	0.440
Leukocytes				
Leukocyte count × 10^3^/mm^3^ #	4.3 (3.2, 0.3–8.9)	3.1 (2, 0.3–8.9)	5.9 (2.1, 3.6–8.7)	0.003 *
Leukopenia, *n* (%)	19 (46%)	17 (63%)	2 (14%)	0.004 *
Severe leukopenia (≥Grade 3), *n* (%)	3 (7%)	3 (11%)	0 (0%)	0.517
Neutrophils				
Neutrophil count × 10^3^/mm^3^ #	1.8 (2.4, 0.1–6.3)	1.2 (1.7, 0.1–6.3)	2.8 (1.7, 1.5–5)	0.009 *
Neutropenia, *n* (%)	22 (54%)	20 (74%)	2 (14%)	<0.001 *
Severe neutropenia (≥Grade 3), *n* (%)	12 (55%)	12 (60%)	0 (0%)	0.104
Lymphocytes				
Lymphocyte count ×10^3^/mm^3^ #	1.1 (0.8, 0.1–3)	1.0 (0.7, 0.1–2.6)	1.8 (1, 0.9–3)	0.003 *
Lymphopenia, *n* (%)	21 (51%)	17 (63%)	4 (28%)	0.037 *
Severe lymphopenia	0	0	0	NA
Platelets				
Platelet count × 10^3^/mm^3^ #	149 (80.2, 14–506)	148 (94.2, 14–506)	162.5 (81.2, 102–252)	0.797
Thrombocitopenia, *n* (%)	22 (54%)	15 (56%)	7 (50%)	0.827
Severe thrombocitopenia (≥Grade 3), *n* (%)	0	0	0	0
ALT, IU #	33 (29.5, 7.0–278.0)	35.5 (56.0, 7.0–278.0)	30.0 (27.7, 12.0–66.0)	0.162
Total bilirubin, mg/dL #	0.5 (0.6, 0.2–2.6)	0.7 (1, 0.3–2.6)	0.5 (0.4, 0.2–1.7)	0.165
GGT, IU	33 (44, 10–1028)	33.5 (44, 10–1028)	47.5 (72.5, 15–365)	0.971
Creatinine, mg/dL #	0.8 (0.27, 0.17–1.4)	0.8 (0.3, 0.1–1.4)	0.8 (0.1, 0.6–1.1)	0.917
AP, mg/dL #	90 (64.0, 44–721)	92.5 (95.2, 44–721)	93.5 (42.2, 71–412)	0.798
Diarrhea, *n* (%)	24 (58%)	21 (78%)	3 (21%)	0.008 *
Severe diarrhea (≥Grade 3), *n* (%)	13 (32%)	13 (48%)	0 (0%)	0.055
Nausea and/or vomiting, *n* (%)	18 (43%)	22 (78%)	2 (14%)	0.023 *
Severe nausea and/or vomiting, (≥Grade 3), *n* (%)	6 (15%)	6 (22%)	0 (0%)	0.209
Mucositis, *n* (%)	14 (34%)	11 (41%)	2 (14%)	0.309
Severe mucositis (≥Grade 3), *n* (%)	4 (10%)	4 (15%)	0 (0%)	0.305
Hand–foot syndrome, *n* (%)	4 (10%)	4 (15%)	0 (0%)	0.331

ALT, alanine aminotransferase; AP, alkaline phosphatase; GGT, gamma glutamyl transferase; IU, international units. # Lowest values were taken during treatment; * *p* value < 0.05.

**Table 3:** Single nucleotide variants in *DPYD* found in the recruited patients.

dbSNP ID	nt Change	aa Change	MAF	ClinVar SIFT PolyPhen-2	Toxicity *n* = 27 (%)	Control *n* = 14 (%)
Toxicity group						
rs61622928	c.1218G>A	p.Met406Ile	0.0003258	Controversial	1 (3.7%)	0 (0.0%)
rs57918000	c.1371C>T	p.Asn457=	0.0001321	Benign	1 (3.7%)	0 (0.0%)
rs17376848	c.1896T>C	p.Phe632=	0.04220	Benign	2 (7.4%)	0 (0.0%)
rs202212118	c.2071G>T	p.Val691Leu	0.0001167	Controversial	1 (3.7%)	0 (0.0%)
	c.2197insA	p.Thr733AsnfsTer14		-	1 (3.7%)	0 (0.0%)
	c*159A>G			-	1 (3.7%)	0 (0.0%)
Toxicity and control groups						
rs1801265	c.85T>C	p.Cys29Arg	0.7755	Benign	7 (25.9%)	3 (21.4%)
rs2297595	c.496A>G	p.Met166Val	0.1018	Controversial	7 (25.9%)	1 (7.1%)
rs56293913	c.1129-15T>C	NA	0.1177	Benign	6 (22.2%)	3 (21.4%)
rs1801158	c.1601G>A	p.Ser534Asn	0.011961	Controversial	3 (11.1%)	1 (7.1%)
rs1801159	c.1627A>G	p.Ile543Val	0.198899	Benign	8 (29.6%)	7 (50%)
rs1801160	c.2194G>A	p.Val732Ile	0.04567	Benign	3 (11.1%)	1 (7.1%)
Control group						
rs116364703	c.1492A>G	p.Gln498=	0.00000776	Benign	0 (0.0%)	1 (7.1%)
rs199469537	c.1524+16C>A	NA	0.0007619	Benign	0 (0.0%)	1 (7.1%)

dbSNP ID, database single nucleotide polymorphism identification number; nt, nucleotide; aa, amino acid; MAF, minor allele frequency in European non-Finnish population in gnomAD; ClinVar, Clinical Variation Consortium; SIFT, Sorting Intolerant From Tolerant; PolyPhen-2, Polymorphism Phenotyping v2. The variant c*159A>G is a new variant of unknown significance placed in the 3′UTR.

## Data Availability

The data are accessible upon request to the corresponding author.

## Supplementary Materials

The following are available online at https://www.mdpi.com/article/10.3390/jpm11080792/s1, Video S1: 3D modeling of p.Thr733AsnfsTer14 truncated and wild type DPD. Table S1: Single nucleotide variants in *DPYD* found in the recruited patients stratified by tumor type.
