# Supplementary material for: DPYD Exome, mRNA Expression and Uracil Levels in Early Severe Toxicity to Fluoropyrimidines: An Extreme Phenotype Approach
Source: J Pers Med. 2021 Aug 13;11(8):792. doi: 10.3390/jpm11080792 (PMC8401253; doi:10.3390/jpm11080792)
Supplement: Supplementary file 1 [file jpm-11-00792-s001.zip › Table S1.pdf]

Table S1. Single nucleotide variants in *DPYD* found in the recruited patients by type of cancer.

dbSNP ID	nt change	aa change	Type of cancer	Toxicity N=27(%)	Control N=14(%)	p value
			Gastric	2 (7.4%)	1 (7.1%)	0.659
rs1801265	c.85T>C	p.Cys29Arg	Colon	3 (11.1%)	0 (0.0%)	0.257
			Rectum	2 (7.4%)	1 (7.1%)	0.809
			Gastric	1 (3.7%)	0 (0.0%)	0.350
rs2297595	c.496A>G	p.Met166Val	Colon	4 (14.8%)	0 (0.0%)	0.081
			Rectum	2 (7.4%)	0 (0.0%)	0.290
			Gastric	1 (3.7%)	0 (0.0%)	0.350
rs56293913	1129-15T>C	NA	Colon	3 (11.1%)	2 (7.4%)	0.920
			Rectum	2 (7.4%)	0 (0.0%)	0.290
			Gastric	2 (7.4%)	0 (0.0%)	0.147
rs1801158	c.1601G>A	p.Ser534Asn	Colon	1 (3.7%)	1 (3.7%)	0.716
			Rectum	0 (0.0%)	0 (0.0%)	na
			Gastric	1 (3.7%)	2 (7.4%)	0.270
rs1801159	c.1627A>G	p.Ile543Val	Colon	4 (14.8%)	4 (14.8%)	0.378
			Rectum	2 (7.4%)	1 (3.7%)	0.809
			Gastric	1 (3.7%)	1 (3.7%)	0.809
rs1801160	c.2194G>A	p.Val732Ile	Colon	0 (0.0%)	0 (0.0%)	na
			Rectum	2 (7.4%)	0 (0.0%)	0.290

dbSNP ID, database single nucleotide polymorphism identification number; nt, nucleotide; aa, aminoacid; na; not applicable.
