# Supplementary figures and images for: DPYD Exome, mRNA Expression and Uracil Levels in Early Severe Toxicity to Fluoropyrimidines: An Extreme Phenotype Approach
Source: J Pers Med. 2021 Aug 13;11(8):792. doi: 10.3390/jpm11080792 (PMC8401253; doi:10.3390/jpm11080792)

## Slide 1
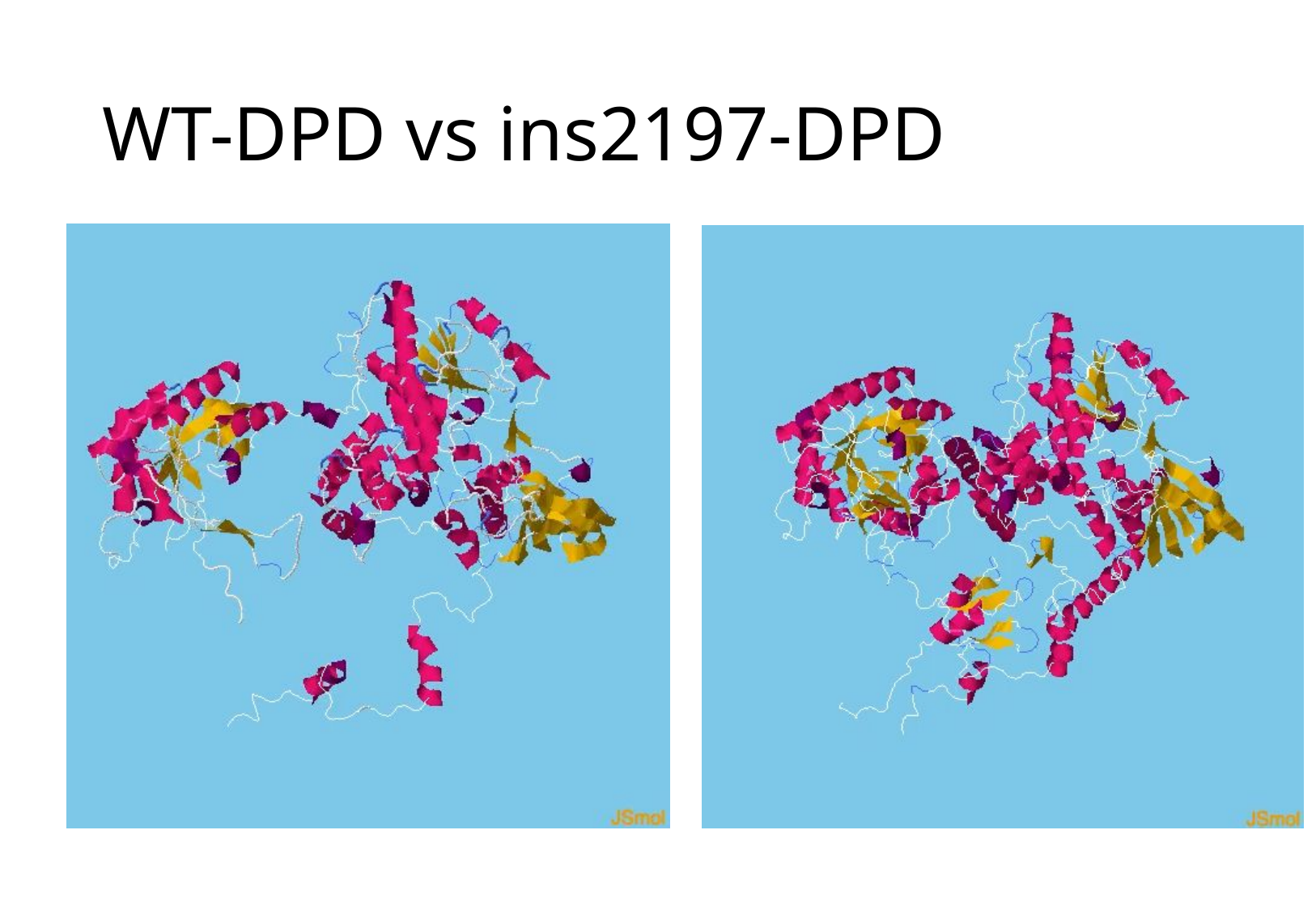

# WT-DPD vs ins2197-DPD

Supplement: Supplementary file 1 [file jpm-11-00792-s001.zip › Video S1.pptx]
